# Magnolol triggers ferroptosis in triple-negative breast cancer through modulation of the ETS2/SLC7A11/GPX4 signaling axis

**DOI:** 10.3389/fphar.2026.1876648

**Published:** 2026-07-31

**Authors:** Jing Zhu, Ke Zhong, Yan Zhuang, Ao Cai, Yu-Han Zhou, Jie Yuan, Feng Ding, Long Zhao, Yu-Xin Zhang, Bo-Han Li, Cheng-Zhu Wu, Hong-Mei Li

**Affiliations:** 1 School of Pharmacy, Bengbu Medical University, Bengbu, China; 2 Anhui Province Biochemical Pharmaceutical Engineering Technology Research Center, Bengbu, China; 3 School of Laboratory Medicine, Bengbu Medical University, Bengbu, Anhui, China

**Keywords:** ETS2, ferroptosis, magnolol, SLC7A11-GPX4, triple-negative breast cancer

## Abstract

**Background:**

Ferroptosis, an iron-dependent form of regulated cell death driven by lipid peroxidation (LPO), represents a promising therapeutic strategy. *Magnolia officinalis*, a traditional herb for resolving dampness and phlegm, is known to modulate cellular metabolism. While magnolol (MAG), a bioactive neolignan from *M. officinalis*, shows anti-TNBC activity, its role in inducing ferroptosis remains unexplored.

**Methods:**

Anti-TNBC effects of MAG were assessed in MDA-MB-231 and 4T1 cells via viability, apoptosis, and ferroptosis assays (intracellular Fe^2+^, LPO, GSH). Target identification employed network pharmacology, RNA-seq, surface plasmon resonance, and pull-down assays. Mechanisms were validated using siRNA/overexpression, Co-IP, and immunofluorescence. *In vivo* efficacy was evaluated in xenograft models.

**Results:**

MAG inhibited proliferation and induced apoptosis and ferroptosis in TNBC cells, evidenced by elevated Fe^2+^ and lipid peroxidation, depleted GSH, downregulated SLC7A11/GPX4, and mitochondrial shrinkage effects reversed by ferroptosis inhibitors. MAG directly bound and suppressed transcription factor ETS2. ETS2 knockdown sensitized cells to MAG-induced ferroptosis, while its overexpression restored SLC7A11/GPX4 expression and conferred resistance. Mechanistically, ETS2 transcriptionally regulated SLC7A11, and MAG enhanced ETS2-SLC7A11 protein interaction. *In vivo*, MAG significantly suppressed tumor growth with low toxicity and downregulated ETS2, SLC7A11, and GPX4.

**Conclusion:**

MAG induces ferroptosis in MDA-MB-231 cells, which may be mediated by targeting the ETS2/SLC7A11/GPX4 signaling axis, providing mechanistic insights into its anti-TNBC activity.

## Graphical Abstract

**Figure:**
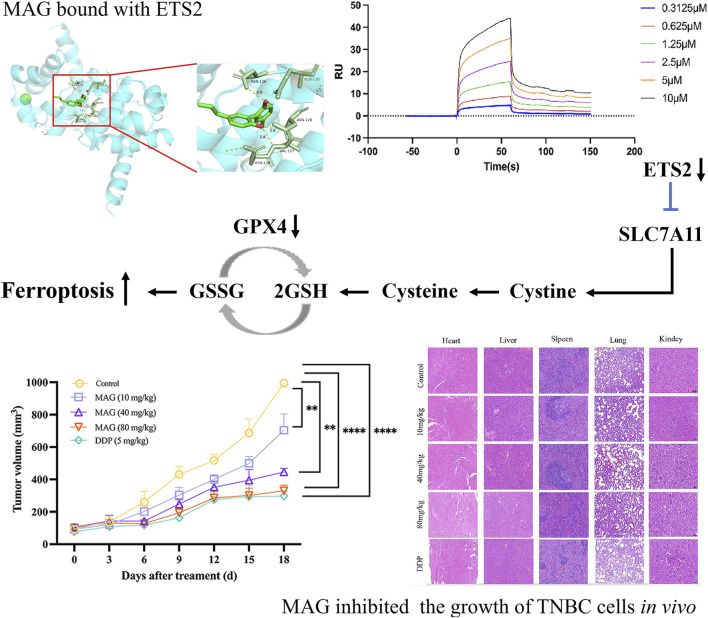

## 1 Introduction

Triple-negative breast cancer (TNBC) represents the most aggressive and lethal subtype of breast cancer. Its poor prognosis and limited treatment options stem from pronounced molecular heterogeneity and the lack of estrogen receptor (ER), progesterone receptor (PR), and human epidermal growth factor receptor-2 (HER-2), with greater severity reported among female patients (Deng et al., 2025; Wu X. et al., 2025). Current treatment strategies primarily include surgery, chemotherapy, immunotherapy, and targeted therapy (Zhao and Qiu, 2025). Although conventional chemotherapy provides clinical benefit for certain TNBC cases, severe toxicity and adverse effects often compromise overall therapeutic efficacy (Liu et al., 2025a). Immunotherapy has introduced new treatment avenues; however, significant drug resistance in TNBC contributes to limited specificity and considerable side effects (Hu et al., 2023). These limitations underscore the pressing need for novel therapeutic targets and more effective treatment options. Natural products have emerged as a promising source of anti-cancer agents, offering low toxicity and high efficacy (Newman and Cragg, 2020).

**FIGURE 1:**
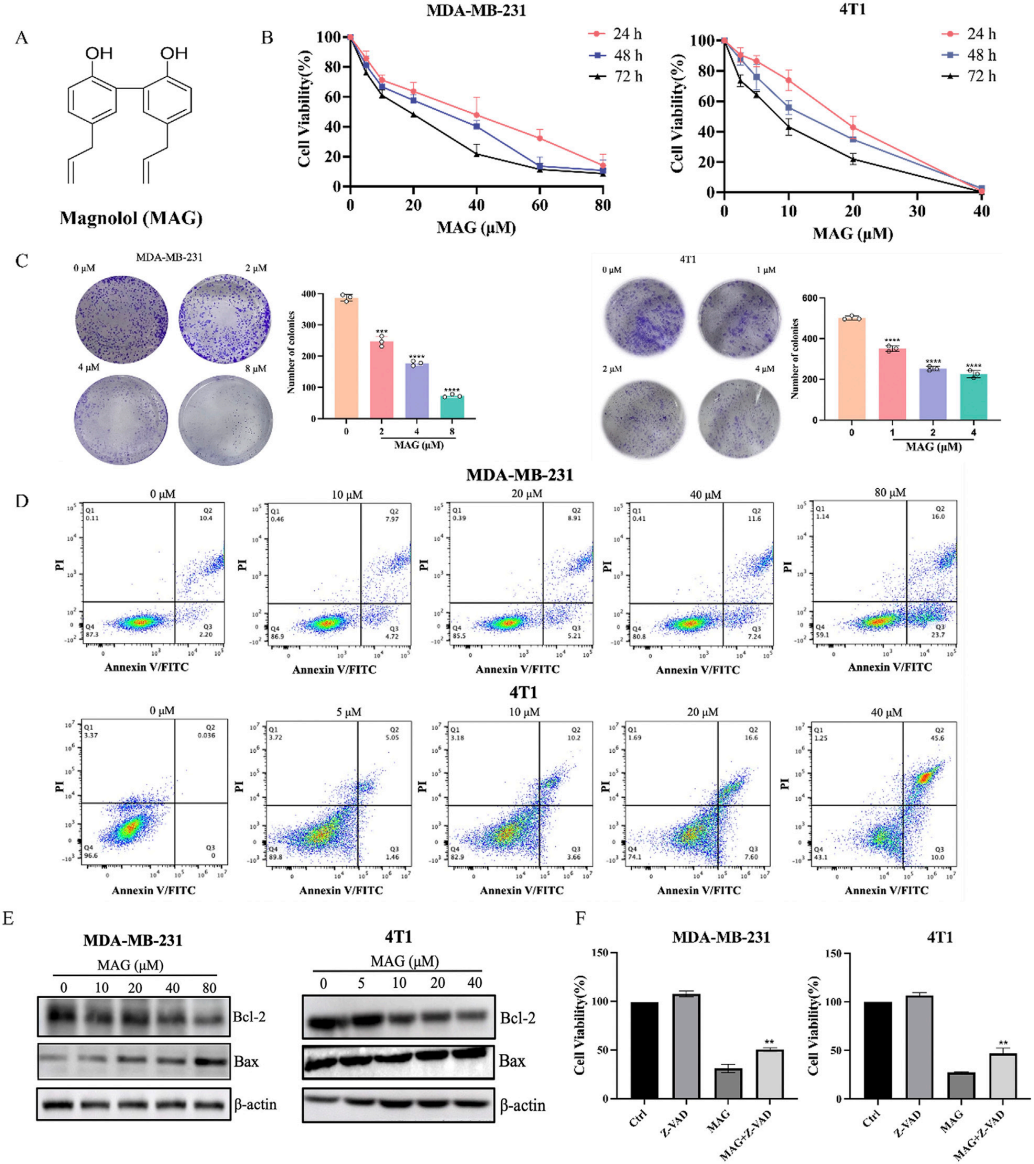
MAG suppresses proliferation and induces apoptosis in TNBC cells **(A)** Chemical structure of MAG **(B)** Cell viability of MDA-MB-231 and 4T1 cells following treatment with various concentrations of MAG by MTT assay **(C)** Colony formation ability of MDA-MB-231 and 4T1 cells after MAG treatment and corresponding quantitative analysis **(D)** Apoptosis detection in MDA-MB-231 and 4T1 cells exposed to differential concentration of MAG for 24 h using Annexin V-FITC/PI double staining **(E)** Western blotting analysis of Bax and Bcl-2 protein expression **(F)** Cell viability analysis by MTT assay in cells treated with MAG (40 μM) for 24 h with or without pre-treatment with z-VAD (20 μM). *p < 0.05, **p < 0.01, ***p < 0.001, and ****p < 0.0001.

Magnolol (MAG, Figure 1A) is a bioactive neolignans isolated from *Magnolia officinalis*, which has potential anti-cancer activities by inhibiting proliferation, inducing apoptosis, suppressing migration and invasion, and anti-angiogenesis in cancer cells (Luo et al., 2019; Wang et al., 2022). However, the precise molecular mechanism and direct targets involved in MAG triggered ferroptosis in cancer cells remain unclear and have yet to be elucidated.

Ferroptosis first described by the Stockwell group in 2012, has played a crucial role in various diseases, such as cancer, neurological disorders, ischemia-reperfusion injury, and liver diseases (Dixon et al., 2012). Increasing evidence suggests that ferroptosis plays a vital role in cancer therapy, with the induction of ferroptosis in tumor cells regarded as an effective strategy to overcome drug resistance (Chen et al., 2025; Dang et al., 2022; Jiang et al., 2024; Zhang et al., 2023). This form of cell death driven by iron-dependent lipid peroxidation (LPO) of polyunsaturated fatty acids abundantly present in the cell membrane (Sokol et al., 2025). GPX4 catalyzes the reduction of phospholipid peroxides to their corresponding phospholipid alcohols, preventing ferroptosis (Schwarz et al., 2023). The inhibition of GPX4 has been shown not only to induce ferroptosis in TNBC but also to increase the efficacy of tumor immunotherapy (Yang et al., 2023).

ETS2, a transcription factor, has been implicated in several diseases, including arteritis, inflammatory bowel disease, primary sclerosing cholangitis, and ankylosing spondylitis (Stankey et al., 2024). While the role of ETS2 in various pathological processes is becoming increasingly recognized (Stankey and Lee, 2025), its relationship with ferroptosis in tumor cells has not yet been elucidated. Investigating the potential connection between ETS2 and ferroptosis in cancer cells may reveal novel therapeutic targets and strategies for the treatment of malignancies and related diseases. This study aimed to investigate the anti-tumor effects of MAG on TNBC and to elucidate the underlying mechanisms, with a particular focus on its ability to induce ferroptosis and its regulation of the ETS2/SLC7A11/GPX4 signaling axis.

## 2 Materials and methods

### 2.1 Materials and reagents

MAG was extracted from *M. officinalis* in our laboratory as previously described. The purity of MAG was determined by high-performance liquid chromatography (HPLC) using a C18 column (Agilent Zorbax SB-C18, 4.6 × 250 mm, 5 μm) with methanol-water (75:25, v/v) as the mobile phase at a flow rate of 1.0 mL/min and detection at 254 nm. The purity was confirmed to be >95%. The MDA-MB-231 and 4T1 cell lines were purchased from the Culture Collection of Chinese Academy of Sciences (Shanghai, China). Ferrostatin-1 (Fer-1) and deferoxamine (DFO) were purchased from MedChemExpress (Shanghai, China). Antibodies against Bax (Cat No.50599-2-Ig), Bcl-2 (Cat No. 12789-1-AP), ETS2 (Cat No.12280-1-AP), and β-actin (Cat No.20536-1-AP) were sourced from Proteintech Group (Wuhan, China). Antibodies against SLC7A11 (HA721868), GPX4 (ab12506), and DMT1 (HA723078) were obtained from Abcam (Cambridge, MA, USA). The iron assay kit (A039-2–1), lipid peroxidation (LPO) assay kit (A106-1–1), and GSH assay kit (A005-1-2) were purchased from Jiancheng Bioengineering Institute (Nanjing, China). The siRNA construct for ETS-2 knockdown human cells was from Sangon Biotech (Shanghai, China).

### 2.2 Cell viability

Cells were maintained in DMEM or RPMI-1640 medium supplemented with 10% fetal bovine serum (FBS) and 1% penicillin/streptomycin in a humidified incubator at 37 °C with 5% CO2. MDA-MB-231 and 4T1 cells were seeded into 96-well plates (a density of 5 × 103 cells/well) and exposed to various concentrations of MAG for 24 h, 48 h, and 72 h. Following the treatment period, 10 μL of MTT solution (5 mg/mL in PBS) was added to each well and further incubated for 4 h in the dark at 37 °C. The culture medium was then carefully removed, and the resulting formazan crystals were dissolved in 100 μL of DMSO per well. The plates were shaken for 10 min at room temperature to ensure complete dissolution. Cells treated with vehicle (0.1% DMSO) served as the control (100% viability), and wells without cells served as blanks. Absorbance (A) was measured at 490 nm using a microplate spectrophotometer (BioTek, USA). Cell viability (%) was calculated as (A_sample_-A_blank_)/(A_control_-A_blank_) ×100% (Pintor et al., 2020).

### 2.3 Colony formation assay

Briefly, MDA-MB-231 and 4T1 cells were cultured in 6-well plates at a density of 1 × 10^3^ cells per well. Once visible colonies had formed, the culture medium was replaced with fresh medium containing low concentrations (1, 2, 4, and 8 μM) of MAG. After 10 days of incubation, the cells were washed with PBS, fixed with 4% paraformaldehyde, stained with 0.1% crystal violet solution, and photographed (Brix et al., 2021).

### 2.4 Flow cytometry with annexin V-FITC/PI staining

Cells were cultured in six-well plates (a density of 5 × 10^5^ cells per well) and incubated overnight to allow for adherence. After incubation, MDA-MB-231 and 4T1 cells were exposed to different concentrations of MAG for 24 h. Following treatment, the cells were harvested, washed three times with PBS, and resuspended in 100 μL of 1× binding buffer. The cells were then stained with 5 μL of Annexin V-FITC and 5 μL of PI (Beyotime, China) for 15 min at room temperature in the dark, followed by the addition of 400 μL of 1× binding buffer. Finally, the percentage of cell death was then analyzed using flow cytometry (Becton-Dickinson, USA) and FlowJo software (Ashland, OR, USA) (Worsley et al., 2022).

### 2.5 Western blotting

Cells were lysed using RIPA buffer (50 mM Tris-HCl pH 7.4, 150 mM NaCl, 1% NP-40, 0.5% sodium deoxycholate, 0.1% SDS) supplemented with 1× protease inhibitor cocktail (Roche, Germany). The lysates were centrifuged at 12,000 × g for 15 min at 4 °C, and the supernatant was collected. Protein concentration was quantified using the bicinchoninic acid (BCA) protein assay kit. Equal amounts of protein were separated by 8%–12% sodium dodecyl sulfate-polyacrylamide gel electrophoresis (SDS-PAGE) and transferred onto polyvinylidene difluoride (PVDF) membranes. The membranes were blocked with 5% skim milk, washed four times with TBS-T buffer, and incubated with the appropriate primary antibodies overnight at 4 °C. After primary, goat anti-rabbit or goat anti-mouse secondary antibodies were applied for 4 h at 4 °C. Finally, protein bands were detected and imaged using a chemiluminescent gel imaging system (Bio-Rad, CA, USA) (Garić et al., 2023).

### 2.6 Measurement of intracellular Fe^2+^, LPO, and GSH levels

Cells were seeded in 6-well plates (a density of 1 × 10^6^ cells per well) and incubated overnight. MDA-MB-231 and 4T1 cells were then treated with MAG or vehicle control, or co-treated with MAG and either Fer-1 or DFO for 24 h. Intracellular levels of Fe^2+^, LPO, and GSH were measured using the corresponding quantification kits. The Fe^2+^ concentration was assessed by measuring the absorbance of the reaction product at 502 nm with a UV spectrophotometer (Thermo Fisher Scientific,USA). LPO and GSH levels were determined by measuring the absorbance at 586 nm and 405 nm, respectively, using a microplate spectrophotometer (BioTek, USA) (Garić et al., 2023; Stockwell, 2022).

### 2.7 Transmission electron microscopy (TEM) analysis

MDA-MB-231 cells were seeded in 6-well plates (a density of 5 × 10^5^ cells per well) and incubated overnight to allow adherence. The cells were then treated with MAG (40 μM) for 24 h. At the end of time point, cells were washed with PBS, fixed with 2% osmium tetroxide, and stained with 1% acetic acid peroxide and 0.1% lead citrate for 12 h. Next, the prepared samples were then sent to Servicebio (Wuhan, China) for TEM analysis (Garić et al., 2023; Peddie et al., 2022).

### 2.8 Transcriptomic analyses performed through RNA sequencing

MAG-treated cells were collected, and 100 μL of RNA extraction reagent was added under light protection conditions. Total RNA was extracted, followed by library preparation and Illumina sequencing. Differential gene expression analysis was performed based on the RNA-Seq data using bioinformatics software. A heatmap of differentially expressed genes was generated using MeV (Multiple Experiment Viewer) software. Functional and classification and pathway enrichment analysis, including KEGG and GO pathways, were conducted using the DAVID (https://davidbioinformatics. nih.gov/summary.jsp). The PPI networks were also constructed using STRING (https://cn.string-db.org/cgi/network) to further explore the interacting proteins and elucidate their biological functions (Garić et al., 2023; Withanage et al., 2022).

### 2.9 Real-time quantitative PCR (RT-PCR)

Total RNA was extracted using the SPARKeasy Tissue/Cell RNA kit, and 1 μg of RNA was used for cDNA synthesis. The RNA concentration and purity were assessed using a Nanodrop ultra-micro spectrophotometer, with 1 μL of each samples measured after calibration with DEPC treated water. Samples showing OD_260/280_ ratios between 1.8 and 2.0 were considered of acceptable quality for further analysis. The cDNA synthesis was performed following instructions of the RevertAid first strand cDNA synthesis kit. Quantitative PCR was carried out using a thermal cycler with the following two-step protocol: initial denaturation at 95 °C for 10 min; followed by 39 cycles of denaturation at 95 °C for 15 s and annealing/extension at 60 °C for 1 min; and a final step at 95 °C for 5 s before holding at 4 °C. The entire process lasted approximately 2 h, after which the data were collected and analyzed (Zucha et al., 2021).

### 2.10 Small interfering RNA (siRNA) transfection

Custom-designed ETS2-specific siRNAs and negative control siRNAs (siNC) were transiently transfected into MDA-MB-231 cells. The siRNA sequence used for ETS2 knockdown was as follows: ETS2 sense, 5ʹ-GGAAGAGUG UCUGGAGCAATT-3ʹ and antisense, 5ʹ- UUG​CUC​CAG​CAG​ACA​UCU​UCC​TT-3ʹ (Friedrich and Aigner, 2022).

### 2.11 Docking results of ETS2 and SLC7A11

The 3D structures of ETS2 (PDB ID:4MHV) and SLC7A11 (PDB ID:7CCS) were retrieved from the Protein Data Bank (https://www.rcsb.org). Structural preparation was performed using AutoDock Tools 1.5.6, including the removal of water molecules and hetero-atoms, addition of polar hydrogens, and assignment of Gasteiger charges. Missing atoms and residues were added using the built-in repair function of AutoDock Tools. For the ligand MAG, its 2D chemical structure was drawn using ChemDraw 20.0 and converted to 3D conformation using ChemDraw 3D, followed by energy minimization with the MM2 force field. Molecular docking was performed using AutoDock Vina 1.1.2 with a grid box centered on the predicted binding pocket of ETS2. The docking results were visualized and analyzed using PyMOL 2.5 to evaluate binding modes, binding energies, hydrogen bonds, and hydrophobic interactions (Eberhardt et al., 2021).

### 2.12 Co-immunoprecipitation assay

A total of 20 μL of Protein A/G magnetic beads were added to each centrifuge tube, followed by the addition of 0.1 μg of rabbit IgG. The mixture was incubated with rotation at 4 °C for 4 h to allow binding. After incubation, unbound material was removed, and the beads washed twice. Following, 400 μL of each protein sample was mixed with 5 μg of flag-ETS2 and incubated at 4 °C for 12 h to form immune complexes. The mixture was then incubated with gentle rotation at room temperature for 4 h, after which the beads were collected using a magnetic rack to remove unbound proteins. The beads were washed four times with 500 μL of RIPA buffer containing protease inhibitors, with the supernatant discarded after each wash. Finally, 100 μL of protein loading buffer was added to the beads, and the mixture was heated at 99 °C for 10 min. The magnetic beads were separated using a magnetic rack, and the supernatant containing the target proteins was collected for further immunoprecipitation and immunoblotting analysis (Tang and Takahashi, 2018).

### 2.13 Small molecule pull down

MDA-MB-231 cells were treated with MAG (40 μM), alongside a control group, and incubated for 24 h with or without biotin-labeled MAG. Following treatment, cells were harvested and lysed in RIPA buffer supplemented with protease and phosphatase inhibitors, followed by brief sonication. The lysates were centrifuged at 12,000 × g for 30 min, and the supernatant was collected. The samples were then incubated overnight at 4 °C with streptavidin beads and biotin-labeled MAG, in the presence or absence of a tenfold excess of unlabeled MAG as a competitive control. After incubation, the beads were washed three times with RIPA buffer, and the bead-bound proteins were eluted, separated by SDS-PAGE, and analyzed by Western blotting (Hwang et al., 2020).

### 2.14 Immunofluorescence staining

MDA-MB-231 cells at approximately 80% confluency were treated with 40 μM of MAG for 24 h. After treatment, the cells were washed three times with PBS, followed by fixation with 1 mL of 4% paraformaldehyde for 30 min at room temperature. The fixed cells were washed three times with PBS (5 min per wash) and permeabilized. Triton X-100 5 min at room temperature, followed by another PBS wash. After permeabilization, the cells were blocked with 3% BSA in PBS containing 0.1% Tween-20 (PBST) for 1 h at room temperature. SLC7A11 (1:200, HA) and ETS2 (1:200, Proteintech) primary antibodies were diluted in 3% BSA blocking solution and incubated with the cells overnight at 4 °C. After three washes with PBST, the cells were incubated with anti-rabbit (1:500, Cat. No. A-21206, Thermo Fisher) and anti-mouse (1:500, Cat. No. A-10521, Thermo Fisher) secondary antibodies for 1 h at room temperature in the dark. After incubation, the cells were washed three times with 1 × TBST. Fluorescently labeled secondary antibodies (FITC or Cy3) were diluted in 3% BSA blocking solution and incubated for 4 h at 4 °C. Following secondary antibody incubation, the cells were washed with 1 × TBST and counterstained with DAPI to visualize nuclei, followed by three PBS washed and final rinse with deionized water. Coverslip edges were sealed with transparent nail polish, and the samples were stored at 4 °C protected from light. Fluorescence images were acquired using a laser confocal microscope (Hwang et al., 2020; Piña et al., 2022).

### 2.15 Surface plasmon resonance (SPR) assay

SPR assay was performed using an SPR biosensor instrument (v. 2.0, Cytiva). ETS2-HA proteins (GeneTex) were immobilized onto a carboxymethylated dextran sensor chip (CM5) via standard amino coupling. To assess the interaction between MAG and ETS2, MAG was injected over the chip surface at a series of concentrations (10, 5, 2.5, 1.25, 0.625, and 0.3125 μM) using PBSP as the running buffer. The sensor surfaces was regenerated with 2 mM NaOH solution between runs. A blank reference channel without ETS2 immobilization was included to account for nonspecific binding and bulk refractive index variations. The binding kinetics and dissociation constants were determined by globally fitting the data to a 1:1 Langmuir binding model using Biacore Insight assessment software (Cytiva, Marlborough, MA, USA) (Sun et al., 2023).

### 2.16 *In vivo* anti-tumor efficacy of MAG

Immunodeficient female BALB/c mice (16–20 g, 4–5 weeks old) were obtained from Qinglongshan (Nanjing, China). All animal procedures were approved by the Ethical Committee of Bengbu Medical University. MDA-MB-231 cells were injected into the right axilla of each mouse in a cross-shaped pattern with a total volume of 200 μL. Once the tumor volume reached approximately 100 mm^3^, the mice were randomly divided into five groups (*n* = 4) and administered intraperitoneal injections of different dose of MAG or DMSO, as follows: (1) Normal control group: 0.1% DMSO; (2) MAG groups: 10 mg/kg; 40 mg/kg; 80 mg/kg; (3) Positive control group: 5 mg/kg cisplatin (DDP). Injections were given every 3 days. Tumor volume and body weight were recorded before each administration. Tumor size was measured using vernier calipers, and tumor volume (V) was calculated with the formula: V (mm^3^) = (length × width^2^)/2. At the end of the experiment, mice were euthanized, tumor and major organs were harvested. Tissues were rinsed with PBS, with portions fixed in 4% paraformaldehyde for histopathological examination by H&E staining, while the remaining tissues were stored at −80 °C for further experiments (Morton and Houghton, 2007).

### 2.17 Statistical analysis

All data were processed, statistically analyzed, and visualized using EXCEL and Prism 9 software. Data are presented as the mean ± standard deviation (SD) from at least three independent experiments. For comparisons between two groups, a two-tailed unpaired Student’s t-test was used. For comparisons involving three or more groups, one-way analysis of variance (ANOVA) followed by Tukey’s *post hoc* test for multiple comparisons was applied. Statistical significance was defined as *p < 0.05, **p < 0.01, ***p < 0.001, and ****p < 0.0001.

## 3 Results

### 3.1 MAG suppresses TNBC cell proliferation and induces apoptosis

MAG exerted significant anti-proliferative effects on MDA-MB-231 and 4T1 cells in a concentration- and time-dependent manner (Figure 1B). Colony formation assays further demonstrated that MAG significantly inhibited cell growth at low concentrations (Figure 1C). Flow cytometry analysis revealed induction of programmed cell death, with apoptosis rates of 12.69%, 14.12%, 18.84%, and 39.70% in MDA-MB-231 cells and 6.51%, 13.86%, 24.20%, and 55.60% in 4T1 cells following MAG treatment at increasing concentrations (Figure 1D). Western blotting analysis confirmed apoptosis induction, as evidenced by an elevated Bax/Bcl-2 radio (Figure 1E). In addition, the application of the pan-caspase inhibitor (z-VAD) reduced MAG-caused cell death (*p* < 0.01) (Figure 1F). These results indicate that MAG effectively inhibits cell proliferation and induces apoptosis in MDA-MB-231 and 4T1 cells.

### 3.2 MAG triggers ferroptosis in TNBC cells

**FIGURE 2:**
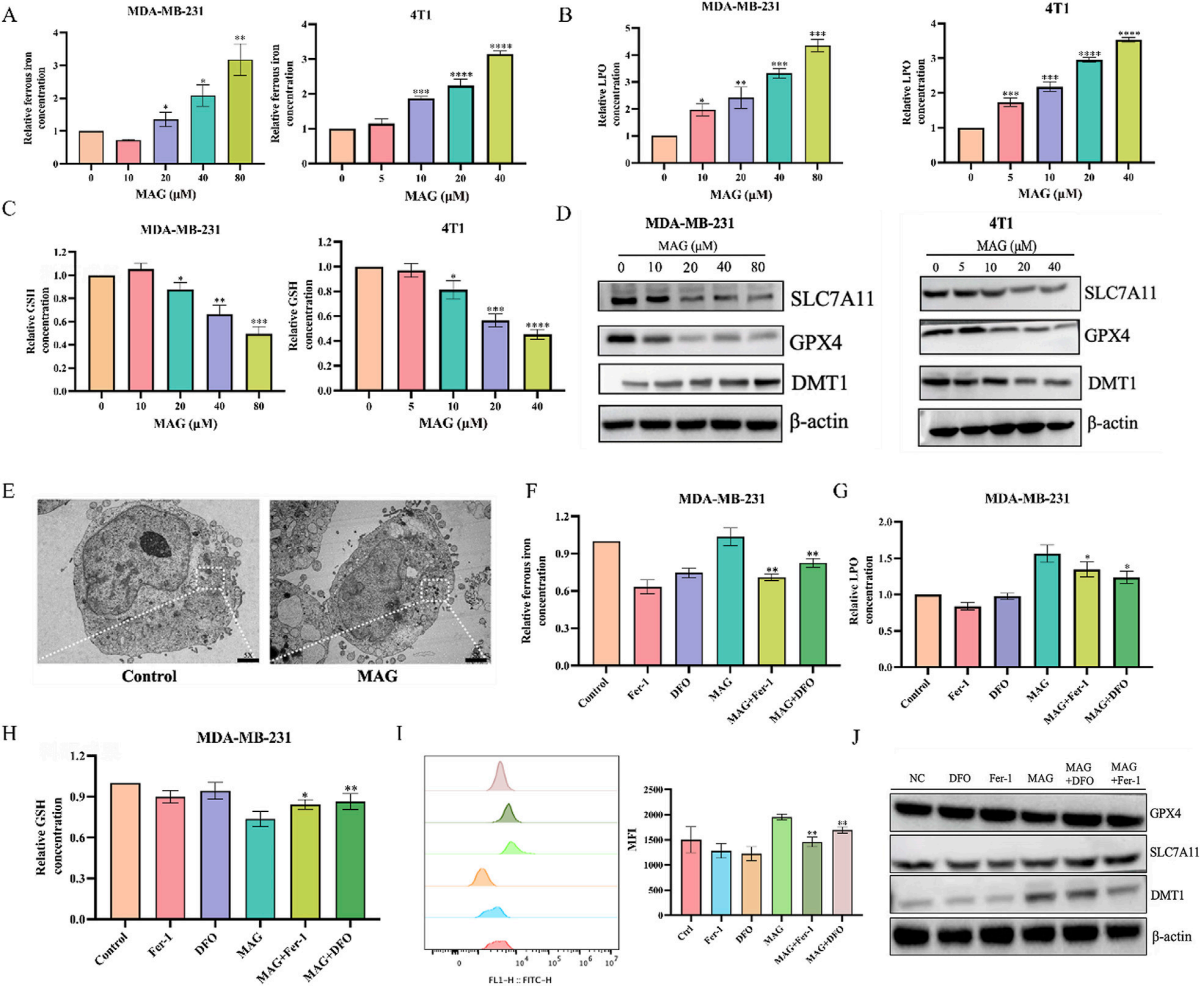
MAG induced ferroptosis in TNBC cells **(A)** Intracellular Fe^2+^ levels in MAG-treated TNBC cells detected using the FerroOrange probe **(B,C)** Measurement of LPO and GSH levels in TNBC cells following MAG treatment **(D)** Protein expression levels of SLC7A11, GPX4, and DMT1 in MAG-treated TNBC cells **(E)** The morphological characteristics was observed by TEM after treatment with MAG (40 μM) for 24 h **(F–I)** Relative levels of Fe^2+^, LPO, GSH, and ROS in MDA-MB-231 cells following 2 h pretreatment with Fer-1 or DFO, followed by MAG exposure **(J)** Western blotting of GPX4, SLC7A11, and DMT1 expression in TNBC cells pretreated with or without Fer-1 or DFO for 2 h, followed by MAG for 24 h *p < 0.05, **p < 0.01, ***p < 0.001, and ****p < 0.0001.

Network pharmacology analysis predicted ferroptosis as a key pathway involved in the anti-tumor activity of MAG against breast cancer cells (Supplementary Figure S1). Previous studies have established that elevated LPO and increased intracellular Fe^2+^ levels are critical events associated with ferroptosis (Yuan et al., 2023). Consistent with this, the present study demonstrated that MAG treatment significantly elevated intracellular Fe^2+^ and LPO levels in MDA-MB-231 and 4T1 cells in a concentration-dependent manner (Figures 2A,B). Furthermore, intracellular GSH level was significantly reduced following MAG exposure (Figure 2C). Western blotting analysis showed that MAG downregulated the expression of SLC7A11 and GPX4, key ferroptosis-related proteins (Figure 2D). Interestingly, we found that the expression of DMT1 protein was opposite in MDA-MB-231 and 4T1 cells. With increasing MAG concentration, the expression level of DMT1 in MDA-MB-231 cells increased, indicating increased sensitivity to ferroptosis. TEM analysis revealed characteristic morphological features of ferroptosis in mitochondria, including reduced mitochondrial volume, loss of cristae, and increased membrane density (Figure 2E). Co-treatment with MAG and ferroptosis inhibitors (Fer-1 or DFO) effectively reversed MAG-induced intracellular Fe^2+^ and LPO levels, GSH depletion, and ROS accumulation (Figures 2F–I). Furthermore, Western blotting demonstrated that these ferroptosis inhibitors significantly restored the expression levels of GPX4, SLC7A11, and DMT1 (Figure 2J). Therefore, these results indicate that MAG induces ferroptosis in TNBC cells.

### 3.3 Transcriptome sequencing analysis indicated that MAG-induced ferroptosis is associated with ETS2 and SLC7A11

**FIGURE 3:**
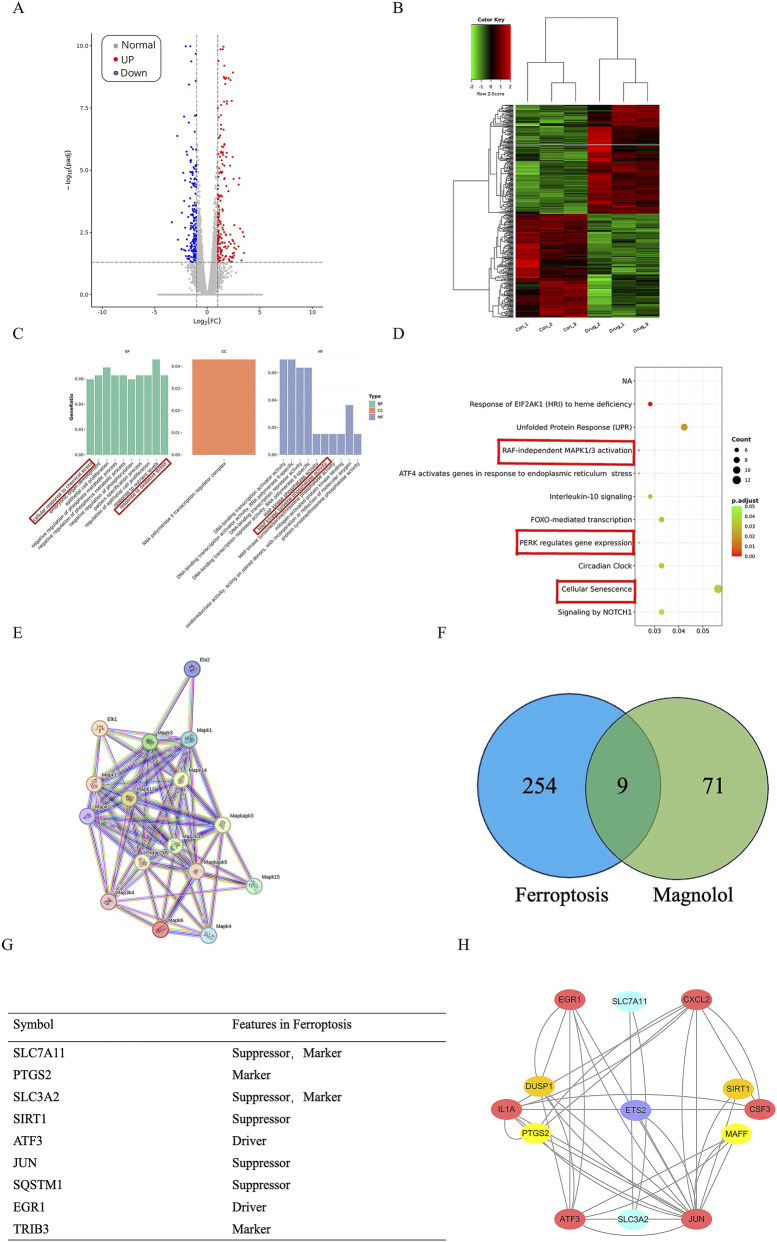
Transcriptome analysis of the effect of MAG in MDA-MB-231 cells **(A)** Volcano plot illustrating upregulated and downregulated genes in MDA-MB-231 cells following MAG treatment **(B)** Heatmap analysis of differentially expressed genes, with red indicating high expression and green indicating low expression **(C)** GO enrichment analysis of differentially expressed genes **(D)** KEGG pathway enrichment analysis **(E–G)** PPI interaction map of ETS2 and differentially expressed genes in MDA-MB-231 cells following MAG treatment **(H)** Intersection analysis showing the regulatory effect MAG on ferroptosis-related genes in MDA-MB-231 cells.

To further explore the anti-cancer mechanisms of MAG, transcriptome sequencing was conducted on MDA-MB-231 cells treated with MAG (40 μM). RNA-seq analysis identified 50 differentially expressed genes following MAG treatment (Figures 3A,B). Pathway enrichment revealed significant involvement of genes associated with oxidative stress response and ferroptosis-related pathways, including *SLC7A11*, *PTGS2*, *JUN*, and *ATF3* (Figures 3C–G). Further protein-protein interaction (PPI) network analysis identified ETS2 as a central regulatory node (Figure 3H). These findings indicate that MAG-triggered cell death is closely linked to ferroptosis and suggest that ETS2 may serve as a potential mediator or target within this process.

### 3.4 ETS2 is identified as a target for MAG induced ferroptosis

**FIGURE 4:**
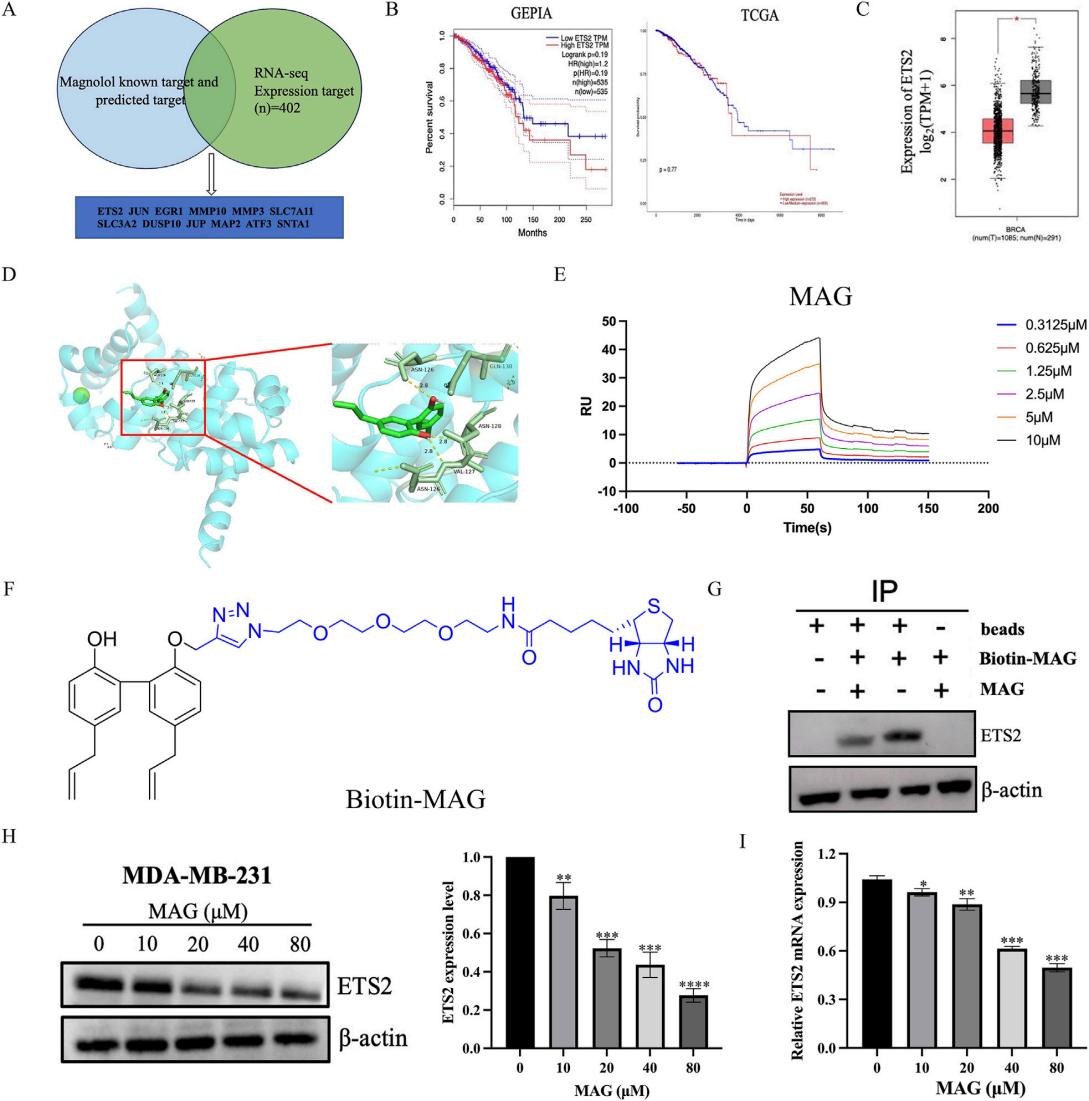
Direct interaction of MAG with the ETS2 protein **(A)** Prediction of potential targets of MAG in MDA-MB-231 cells **(B,C)** Bioinformatics analysis of ETS2 in breast cancer cells **(D)** Molecular docking model illustrating the binding interaction between MAG and the crystal structure of ETS2 (PDB code: 4mhv) **(E)** SPR analysis assessing the binding affinity of MAG to ETS2 **(F)** Chemical structure of biotin conjugated MAG **(G)** Assessment of ETS2 protein expression following treatment with Biotin-MAG **(H)** Effects of MAG on ETS2 protein levels in MDA-MB-231 cells **(I)** Effects of MAG on ETS2 mRNA expression in MDA-MB-231 cells.

To further validate the target of MAG and clarify potential intersecting targets based on transcriptome sequencing, RNA-seq analysis of MAG-treated MDA-MB-231 cells identified ETS2 as a differentially expressed gene (Figure 4A). In parallel, survival analysis using TCGA and GEPIA databases revealed that elevated ETS2 expression associated with poorer overall survival in breast cancer patients (Figures 4B,C). Molecular docking predicted a stable interaction between MAG and the ETS2 binding domain, with a binding energy of −6.9 kcal/mol and hydrogen bonds formed with ASN126, ASN128, and VAL127 (Figure 4D). SPR assay further confirmed the direct binding of MAG to ETS2, with an estimated K_d_ value of 3.62 μM (Figure 4E).

To experimentally confirm this interaction, a biotin-labeled MAG probe was synthesized and used in a small-molecule pull-down assay (Supplementary Scheme S1). The assay demonstrated specific binding of biotin-MAG to endogenous ETS2 in cell lysates, which was abolished by competition with excess unlabeled MAG, confirming the specificity of the interaction (Figures 4F,G; Supplementary Figure S2). Western blotting and qPCR experiments showed that MAG reduced ETS2 expression in MDA-MB-231 cells (Figures 4H,I). Immunofluorescence further confirmed reduced nuclear ETS2 following MAG treatment (Supplementary Figure S3). These findings indicate that ETS2 is a direct molecular target of MAG in inducing ferroptosis in MDA-MB-231 cells.

### 3.5 MAG inhibits ETS2 and activates ferroptosis by decreasing SLC7A11

**FIGURE 5:**
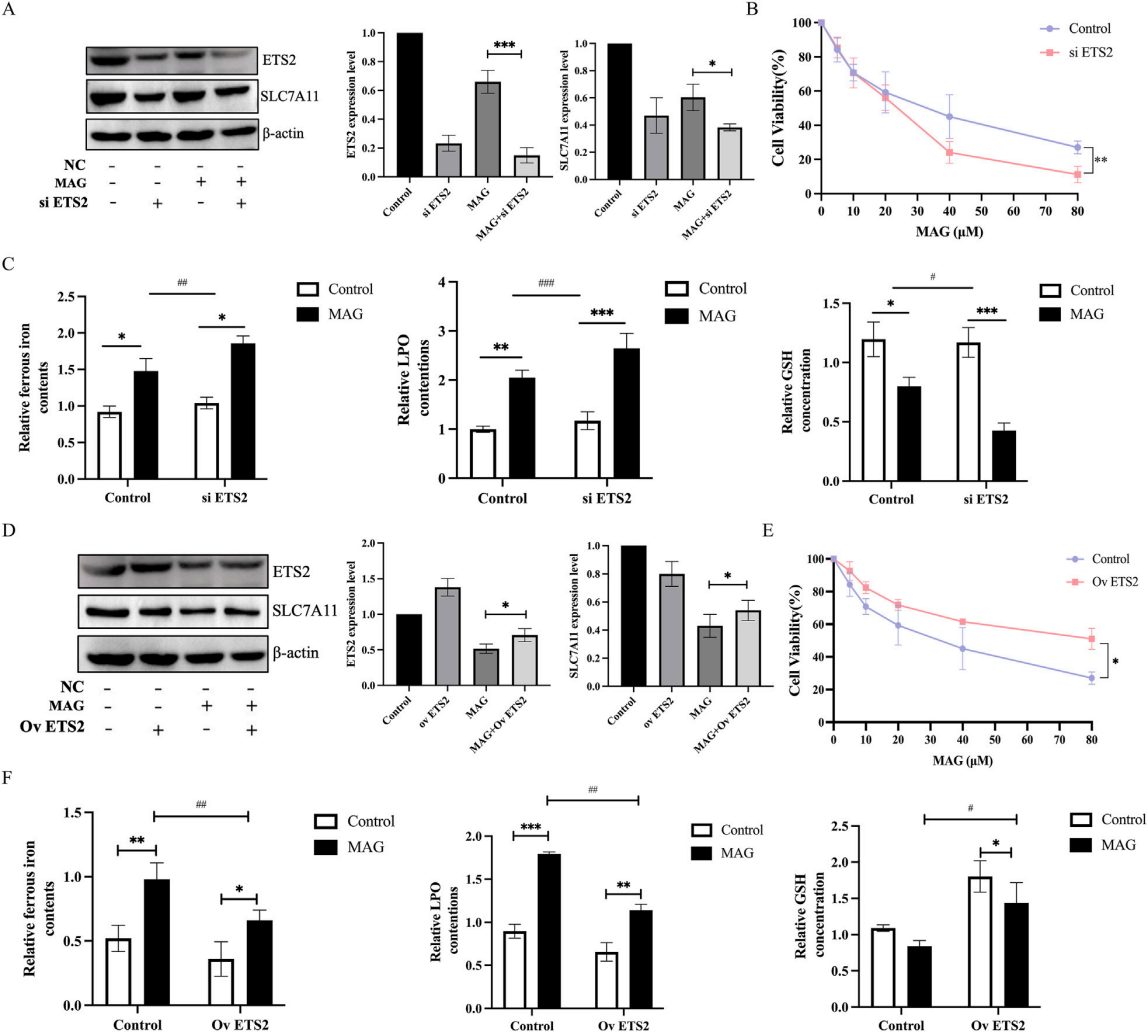
MAG induced ferroptosis in MDA-MB-231 cells through ETS2 suppression **(A)** Western blot analysis confirming the knockdown efficiency of ETS2 and corresponding changes in SLC7A11 protein expression **(B)** Cell viability following knockdown of ETS2 in MDA-MB-231 cells **(C)** Effects of ETS2 knockdown on intracellular Fe2+, LPO, and GSH levels **(D)** Western blotting confirming ETS2 overexpression and its effect on SLC7A11 protein levels **(E)** Cell viability following overexpression of ETS2 in MDA-MB-231 cells **(F)** Effects of ETS2 overexpression on intracellular Fe^2+^, LPO, and GSH levels. *p < 0.05, **p < 0.01 and ***p < 0.001 compared with the control. #*p* < 0.05, ##*p* < 0.01, and ###*p* < 0.001 compared with no overexpression.

The siRNA transfection and ETS2 plasmid transfection experiment were also performed in this study. Western blotting analysis revealed that knockdown of ETS2 by siRNA led to a reduction in both ETS2 and SLC7A11 protein levels (Figure 5A). As shown in Figure 5B, ETS2 knockdown significantly reduced cell viability compared to the control group. Moreover, siRNA-mediated silencing of ETS2 further elevated intercellular Fe^2+^ accumulation, LPO levels, and GSH depletion following MAG (40 μM) treatment in MDA-MB-231 cells (Figure 5C). ETS2 overexpression via plasmid transfection restored SLC7A11 protein expression (Figure 5D). As shown in Figure 5E, ETS2 overexpression significantly increased cell viability. Furthermore, ETS2 overexpression significantly reduced intracellular Fe^2+^ and LPO levels, and restored GSH levels in MAG treated cells (Figure 5F). Taken together, these results indicate that MAG induces ferroptosis in MDA-MB-231 cells by targeting ETS2.

### 3.6 MAG enhances the interaction between ETS2 and SLC7A11 protein interaction

**FIGURE 6:**
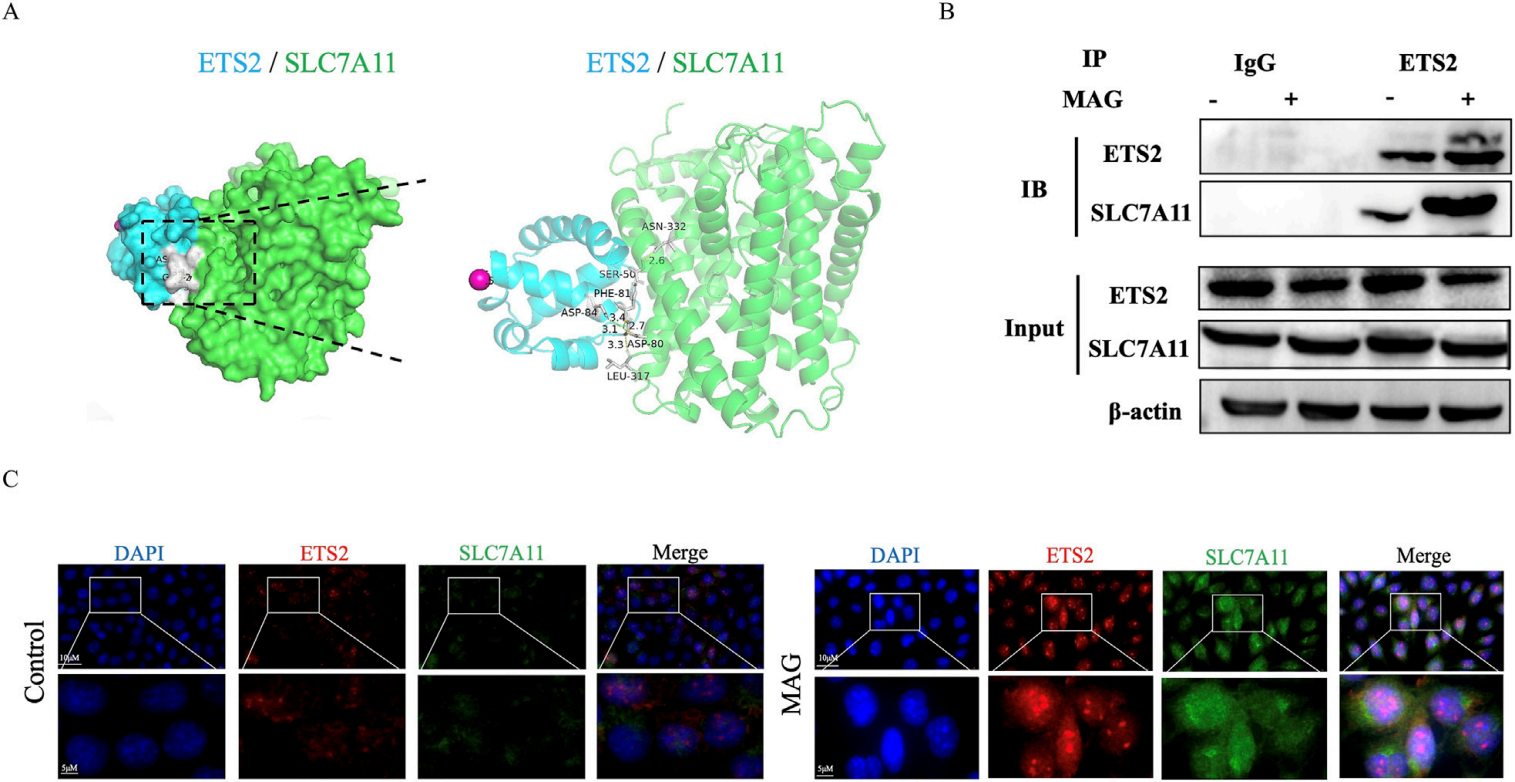
MAG promotes the interaction between ETS2 and SLC7A11 **(A)** Molecular docking model illustrating the interaction between ETS2 and SLC7A11 proteins **(B)** Western blotting confirming the interaction between ETS2 and SLC7A11 proteins **(C)** Immunofluorescence assay visualizing the interaction between ETS2 and SLC7A11 proteins.

Transcriptome analysis suggested a potential interaction between ETS2 and SLC7A11 proteins. Molecular docking revealed that ETS2 can directly bind to SLC7A11, with a binding energy of −25.9 kcal/mol (Figure 6A). This interaction was further confirmed by co-immunoprecipitation, which demonstrated a direct association between ETS2 and SLC7A11 (Figure 6B). MAG treatment appeared to increase this protein interaction. In MDA-MB-231 cells, reduced nuclear co-localization of ETS2 (red) and SLC7A11 (green) was observed under basal conditions, whereas MAG treatment resulted in increased co-localization of ETS2-SLC7A11 signals (Figure 6C). These findings indicate that MAG promotes the interaction between ETS2 and SLC7A11 proteins.

### 3.7 MAG inhibits tumor growth and shows favorable safety

**FIGURE 7:**
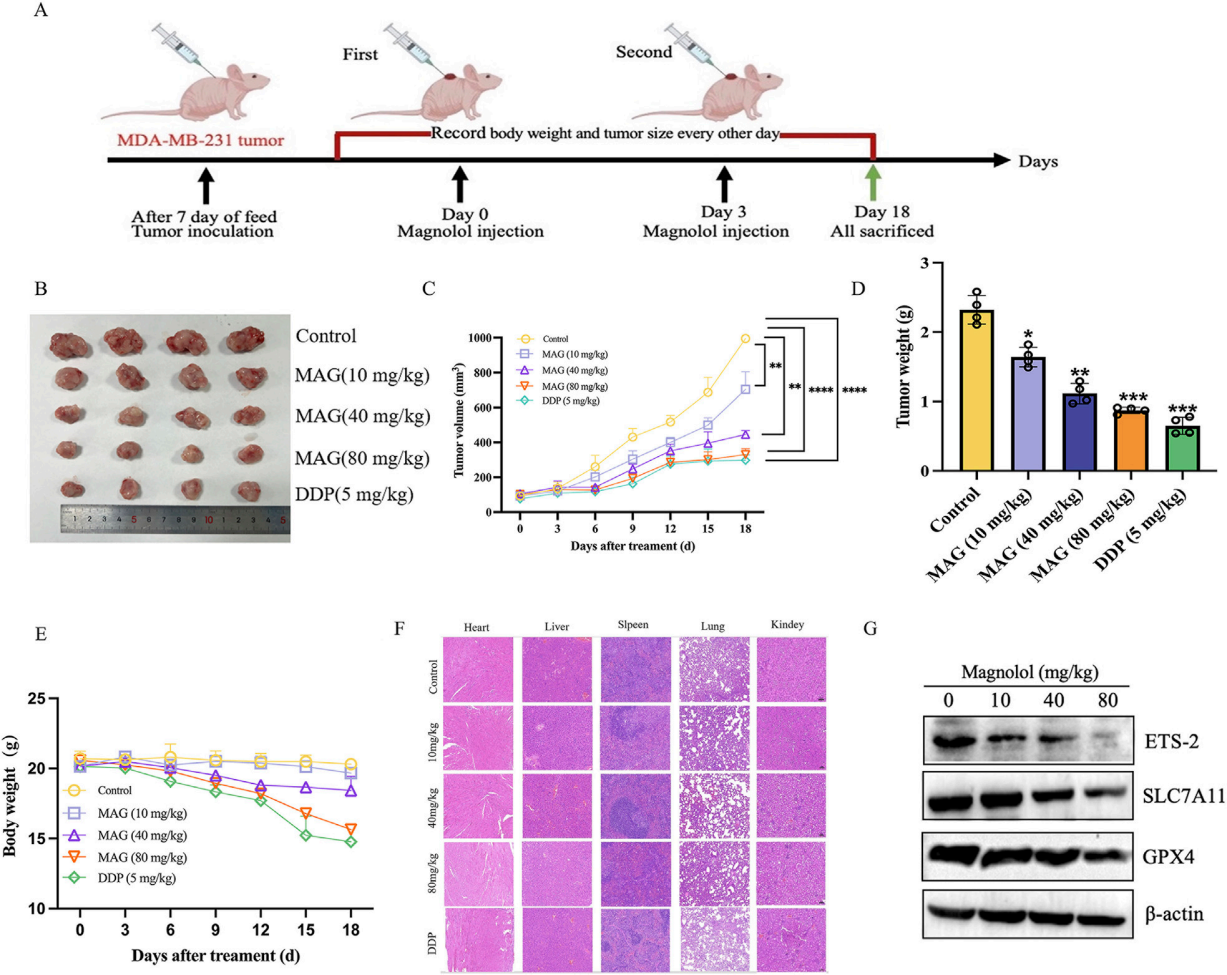
MAG inhibits the growth of MDA-MB-231 cells *in vivo* (n = 4) **(A)** Experimental scheme for subcutaneous tumor implantation and drug administration in nude mice **(B)** Representative images of excised tumor tissues from mice implanted with MDA-MB-231cells **(C–E)** Quantitative analysis of tumor volume growth curves, body weight, and tumor final weight each group **(F)** H&E staining of the heart, liver, spleen, lung, and kidney to assess potential tissue toxicity **(G)** Western blot analysis of ETS2, SLC7A11, and GPX4 protein levels in tumor tissues. *p < 0.05, **p < 0.01, ***p < 0.001, and ****p < 0.0001 compared with the control.

MDA-MB-231 cell xenograft models were established to assess the *in vivo* anti-tumor efficacy of MAG (Figure 7A). As shown in Figures 7B–D, MAG significantly suppressed tumor growth in a dose-dependent manner. Compared with the vehicle control group, MAG administration at doses of 10, 40, and 80 mg/kg inhibited tumor growth by 35.27%, 54.26%, and 64.73%, respectively. The positive control drug, DDP suppressed tumor growth by 78.68% in tumor volume. No significant body weight loss and signs of organ damage were observed following MAG treatment (Figures 7E,F). Western blotting analysis of tumor tissues revealed a significantly reduction in ETS2, SLC7A11, and GPX4 protein levels in response to MAG treatment (Figure 7G). Taken together, these results indicate that MAG induces ferroptosis in MDA-MB-231 cells by targeting ETS2.

## 4 Discussion

TNBC remains a major clinical challenge due to its significant heterogeneity, high malignancy, aggressive progression, and high recurrence rates, which contribute to elevated breast cancer-related mortality (Cheng et al., 2024). The lack of effective targeted therapies underscores the urgent need to identify safe and potent novel agents. Natural products, known for their low toxicity and multi-targeting properties, have gained attention as promising candidates for cancer treatment, particularly through the induction of regulated cell death (RCD) pathways such as ferroptosis (Zhang et al., 2024). Ferroptosis, an iron-dependent form of RCD driven by LPO, has emerged as a potential therapeutic strategy for TNBC, especially for subtypes resistant to conventional apoptosis based treatments (Gong et al., 2023; Wu Z. et al., 2025).

MAG, a bioactive polyphenolic compound isolated from *Magnolia officinalis*, shows a wide range of pharmacological properties, including well established anti-tumor activity (Qiao et al., 2023). In the present study, MAG exerted significant anti-TNBC effects both *in vitro* and *in vivo* by effectively inhibiting tumor cell proliferation and inducing significant cytotoxicity. Additionally, MAG significantly enhanced the efficacy of paclitaxel and doxorubicin in MDA-MB-231 cells, with combination index (CI) values <1 (Supplementary Figure S4). This study provides the first evidence that MAG induces cell death in TNBC cells predominantly through a ferroptosis-dependent mechanism.

To elucidate the molecular mechanism underlying MAG-induced ferroptosis in TNBC, the present study focused on the transcription factor ETS2. As a key member of the ETS family, ETS2 contains transcriptional activation and DNA-binding domains and plays essential roles in regulating inflammation, tumorigenesis, and cancer progression (Choi et al., 2023; Vázquez-Cabrera et al., 2024). The elevated ETS2 expression is associated with increased tumor malignancy, which its downregulation significantly suppresses cancer cell proliferation and invasion, partly by modulating p16^INK4a^ expression through the Ras pathway (Hume et al., 2025; Li et al., 2011; Zhang et al., 2018). The present findings identified a novel mechanism central to MAG’s activity, demonstrating that MAG directly binds to ETS2 with high affinity. This interaction was confirmed by molecular docking, pull-down assays, and SPR analysis. Moreover, ETS2 knockdown significantly increased MAG-induced ferroptosis, as reflected by increased ferroptosis-related markers. These results establish ETS2 as a direct functional target of MAG mediating its ferroptotic effects in TNBC cells.

The downstream pathway connecting ETS2 to ferroptosis regulation was further investigated. The core regulators of ferroptosis resistance are the cystine/glutamate antiporter SLC7A11 (a component of system Xc^−^) and the selenoprotein GPX4. SLC7A11 and GPX4 are critical regulators of ferroptosis and have been proposed as potential predictive and prognostic biomarkers for breast cancer patients undergoing neoadjuvant chemotherapy (Wu et al., 2024). SLC7A11, frequently upregulated in cancer cells under oxidative, metabolic, or amino acid stress, maintains intracellular redox homeostasis by facilitating cystine uptake for GSH synthesis, protecting against ferroptosis (Hill and Liu, 2022; Koppula et al., 2018; Wang et al., 2023; Wang et al., 2024). GPX4, the primary enzyme using GSH, detoxifies lipid hydroperoxides by converting them to lipid alcohols, preventing the iron-dependent accumulation of lethal lipid reactive oxygen species (ROS) that drive ferroptosis (Beatty et al., 2021; Ferreira et al., 2023; Xia et al., 2024; Xue et al., 2025).

Inhibition of either SLC7A11 or GPX4 is a well-established approach to induce ferroptosis in tumor cells (Liu et al., 2025b; Peng and Mo, 2022). This study demonstrated that MAG treatment significantly enhanced the interaction between ETS2 and SLC7A11 proteins. This increased interaction was accompanied by a significant downregulation of both SLC7A11 and GPX4 protein expression in TNBC cells. These findings indicate that MAG, by targeting ETS2, promotes the formation of an ETS2-SLC7A11 complex resulting in suppression of the SLC7A11/GPX4 axis. Disruption of this axis compromises the cellular defense against LPO, ultimately inducing ferroptosis in TNBC. ETS2 transcriptionally regulates SLC7A11, as supported by loss- and gain-of-function expression data and bioinformatic correlation; direct binding of ETS2 to the SLC7A11 promoter remains to be established with ChIP-qPCR or luciferase assays, which will be pursued in future studies.

The translational relevance of our *in vitro* findings is supported by the *in vivo* xenograft experiments using immunodeficient BALB/c (nude) mice bearing MDA-MB-231 tumors. The MDA-MB-231 cell line is a widely accepted model for TNBC due to its triple-negative phenotype (ER^−^/PR^−^/HER2^-^) and high invasive capacity, which closely mirrors the aggressive clinical behavior of human TNBC. Notably, the dose-dependent anti-tumor effect of MAG observed *in vivo* (10, 40, and 80 mg/kg) correlated well with the concentration-dependent ferroptosis induction seen *in vitro*. Furthermore, the downregulation of ETS2, SLC7A11, and GPX4 in tumor tissues from MAG-treated mice (Figure 7G) directly mirrors the protein expression changes observed in cultured MDA-MB-231 cells (Figures 2D,J), confirming that the ferroptosis pathway is operational in the *in vivo* setting. Collectively, the consistency between the cellular and animal models strengthens the conclusion that MAG exerts its anti-TNBC activity through the ETS2/SLC7A11/GPX4 axis.

**FIGURE 8:**
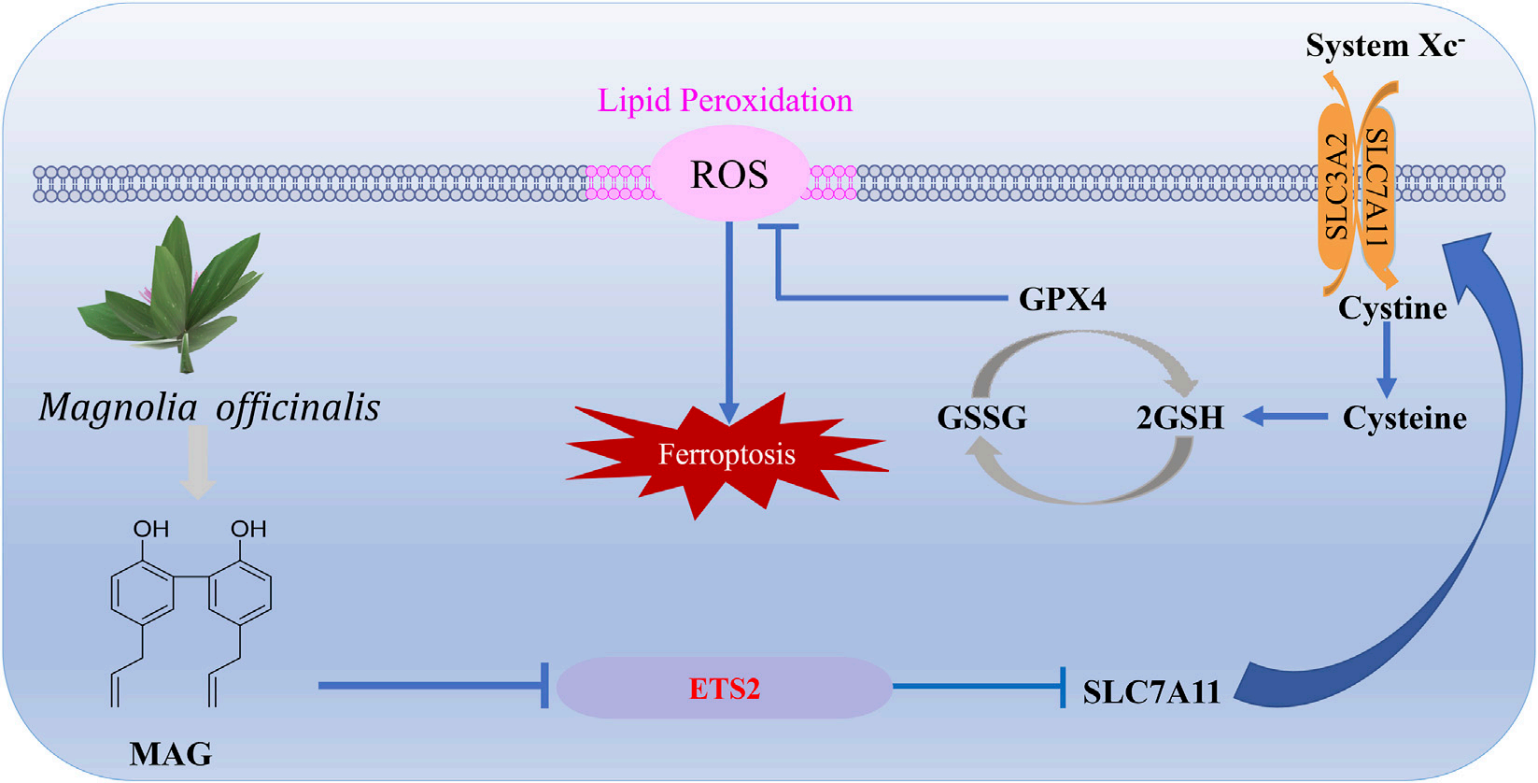
Schematic illustration of MAG triggered ferroptosis via the ETS2/SLC7A11/GPX4 signaling axis in MDA-MB-231 cells.

In conclusion, the findings indicate that ETS2 serves as a pivotal molecular target mediating the ferroptosis-inducing and anti-TNBC effects of MAG. MAG directly binds to ETS2, enhancing its association with SLC7A11 and suppressing the expression of GPX4 (Figure 8). This study identifies a novel regulatory mechanism by which MAG inhibits TNBC cell growth through the ETS2/SLC7A11/GPX4 signaling axis, highlighting the potential of MAG as a potential candidate for TNBC treatment.

## Data Availability

The original contributions presented in the study are publicly available. The RNA-sequencing data have been deposited in the Gene Expression Omnibus (GEO) repository under accession number GSE341147. All other raw data supporting the findings of this study are included in the article and Supplementary Material. For any inquiries regarding the data, please contact the corresponding authors.
