# Supplementary material for: Magnolol triggers ferroptosis in triple-negative breast cancer through modulation of the ETS2/SLC7A11/GPX4 signaling axis
Source: Front Pharmacol. 2026 Jul 31;17:1876648. doi: 10.3389/fphar.2026.1876648 (PMC13473434; doi:10.3389/fphar.2026.1876648)
Supplement: Supplementary file 1 [file Supplementaryfile1.docx]

Supplementary Data

**Table of contents**

**SCHEME S1.** Chemical synthesis of biotin-MAG	3

**FIGURE S1.** Network pharmacology predicts potential targets of MAG in the treatment of breast cancer.	4

**FIGURE S2.** The functions of biotin-MAG	5

**FIGURE S3.** Immunofluorescence assay	5

**FIGURE S4.** The combined effect of MAG and doxorubicin or paclitaxel	6

**FIGURE S5−S7.** Compound characterization spectra	7

**Materials and methods**	9

**Synthetic procedures and characterized data of MAG/biotin compound***…...……*9

**Network pharmacology prediction assay**…………………………………………10

**Measurement of combination index (CI)***………………………………………………*10

**SCHEME S1** **|** Chemical synthesis of biotin-MAG.

Reagents and conditions: (a) Propargyl bromide, K_2_CO_3_, DMF, r.t.; (b) Biotin-PEG, anhydrous copper sulfate, sodium ascorbate, tert-butanol: H_2_O = 1:1, r.t..

**FIGURE S1**

Network pharmacology predicts potential targets of MAG in the treatment of breast cancer. (**A**) Venn diagram of the intersection of the targets of MAG and the targets related to breast cancer. (**B,C**) Analysis of protein interactions between MAG and potential intersectional targets for breast cancer. (**D**) GO enrichment analysis. (**E**) KEGG analysis. (**F**) The network diagram of "Drug-Target-Disease".

**FIGURE S2**

The functions of biotin-MAG. (**A**) Cell viability of Biotin-MAG in MDA-MB-231 cells. (**B–D**) Fe^2+^, LPO and GSH levels in Biotin-MAG treated MDA-MB-231 cells were assayed.

**FIGURE S3**

Immunofluorescence assay.

**FIGURE S4****.**

The combined effect of MAG and doxorubicin or 9aclitaxel. (**A**) The Chou-Talalay method confirmed the synergistic effect of MAG and doxorubicin. (**B**) The Chou-Talalay method confirmed the synergistic effect of MAG and paclitaxel.

**FIGURE S5**

The ^1^H NMR spectra (400 MHz) of compound biotin-MAG in CDCl_3_.

**FIGURE S6**

The ^13^C NMR spectra (100 MHz) of compound biotin-MAG in CDCl_3_.

**FIGURE S7**

The HR-ESI-MS spectrum of biotin-MAG.

**Materials and methods**

**Synthetic procedures and characterized data of MAG/biotin compound**

MAG (0.26 g, 1 mM) was dissolved in anhydrous DMF (3 mL), propargyl bromide (0.13 g, 1.1 mM) and potassium carbonate (0.27 g, 2 mM) were added, and the reaction was stirred at room temperature for 8 h. After the reaction was monitored by TLC, the reaction system was quenched by adding water to the reaction system, extracted with ethyl acetate and water, dried with anhydrous sodium sulfate, and spun dry. The petroleum ether:ethyl acetate (10:1) eluate was eluted by column chromatography (300-400 mesh) to give intermediate 1, a yellow oil with 47 % yield. Intermediate 1 (0.06 g, 0.21 mM) was dissolved in tert-butanol:water = 1:1 (1 mL), and biotin-PEG (0.044 g, 0.1 mM), anhydrous copper sulfate (0.003 g, 0.021 mM), and sodium ascorbate (0.004 g, 0.021 mM) were added, and the reaction was stirred at room temperature for 8 h. The end of the reaction was monitored by TLC, and then the reaction was eluted with dichloride After TLC monitoring, the reaction was eluted with dichloride and spun dry. Dichloride: methanol = 10:1 climbed the large plate to get the total product Biotin-MAG, yellow oil, yield 21%. ^1^H NMR (400 MHz，CDCl_3_) *δ* 7.66 (s，1H)，7.18 – 7.10 (m，2H)，7.10 – 7.00 (m，3H)，6.92 (d，*J* = 8.1 Hz，1H)，6.66 (t，*J* = 5.5 Hz，1H)，6.54 (s，1H)，5.96 (tt，*J* = 16.7，6.6 Hz，2H)，5.60 (s，1H)，5.22 (s，2H)，5.13 – 5.00 (m，4H)，4.49 (s，2H)，4.25 (t，*J* = 6.4 Hz，1H)，3.83 (t，*J* = 5.1 Hz，2H)，3.55 (s，4H)，3.50 (d，*J* = 2.8 Hz，4H)，3.47 (s，2H)，3.36 (d，*J* = 7.4 Hz，4H)，3.32 (d，*J* = 14.1 Hz，2H)，3.07 (d，*J* = 12.1 Hz，1H)，2.83 (d，*J* = 8.2 Hz，1H)，2.67 (d，*J* = 12.6 Hz，1H)，2.09 (t，*J* = 7.5 Hz，2H)，1.71 – 1.54 (m，4H)，1.26 (s，2H)，0.91 – 0.85 (m，2H); ^13^C NMR (400 MHz，CDCl_3_) *δ* 173.57，164.09，153.22，152.21，143.82，137.85，137.39，133.99，132.28，131.84，131.38，129.01，128.23，126.16，124.09，116.80，115.91，115.53，113.78，70.58，70.29，69.83，69.35，63.44，61.78，60.23，55.58，50.31，40.48，39.43，39.40，38.93，35.82，31.60，28.21，28.06，25.66，22.67，14.15; HRMS Calculated for C_39_H_53_N_6_O_7_S *m/z* 748.3618，found *m/z* 749.3633.

**Network pharmacology prediction assay**

On the TCMSP platform, the chemical structure of MAG was queried, and the InChIKey was obtained. Then, the Canonical SMILES number was retrieved from the PubChem website and all proteins were downloaded in the Potential Targets column. The potential targets of MAG were predicted through the Swiss Bioinformatics Institute (Swiss, http://swisstargetprediction.ch/). Subsequently, the results from the two databases were combined and the repetitive parts were removed. The protein targets were converted into gene targets using the UniProt database (<http://www.uniprot.org/uniport>). The MeSH name Breath Neoplasms was obtained by inputting Breath Cancer into the MeSH database, and the related disease targets of breast cancer were predicted in Genecards database, OMIM and TTD database. The obtained drug targets and breast cancer targets were plotted using the online software Jven (https://jvenn.toulouse.inrae.fr/app/example.html) to obtain their intersection. The intersection genes were analyzed using the DAVID online software (https://david.ncifcrf.gov/summary.jsp) for gene GO and KEGG analysis and visualized using the MicrobiomeNet online analysis and visualization cloud platform (http://www.bioinformatics.com.cn) for visualization analysis. Using the Venny 2.1.0 online website, we identified the intersection targets of MAG and breast cancer. These intersection targets have the potential to become the targets for MAG in treating breast cancer. Subsequently, the relevant information of drugs, diseases and intersection targets was imported into the Cytoscape 3.7.2 software to construct a "drug-target-disease" network diagram. The network diagram consists of multiple nodes, each representing a drug, intersection target or disease, while the edges represent the connections between them.

**Measurement of combination index (CI)**

According to the Chou-Talalay method, the concentration of the administered drug and the concentration of the administered drug alone and in combination were set first. The specific groupings were as follows: (1) Paclitaxel: (5, 10, 20, 40, and 80 μM); Adriamycin: (2.5, 5, 10, 20, and 40 μM) to be incubated at the end. Processing and calculations were carried out according to the subsequent processing steps of the MTT experimental operation. The obtained drug concentrations and drug inhibition rates were entered into the software accompanying the Chou-Talalay method, and the drug combination indices (CI) values at different concentrations were automatically calculated according to the steps and operational procedures in the software.
